# Human Hantavirus Infections in Hungary (2018–2025): Epidemiology, Molecular Detection Across Clinical Sample Types, and Phylogenetic Analysis

**DOI:** 10.3390/v18030366

**Published:** 2026-03-16

**Authors:** Anita Koroknai, Anna Nagy, Orsolya Nagy, Nikolett Csonka, Levente Zsichla, Katalin Szomor, Mária Takács

**Affiliations:** 1 National Reference Laboratory for Viral Zoonoses, National Center for Public Health and Pharmacy, 1097 Budapest, Hungary; nagy.anna@nngyk.gov.hu (A.N.); nagy.orsolya@nngyk.gov.hu (O.N.); csonka.nikolett@nngyk.gov.hu (N.C.);; 2 Institute of Medical Microbiology, Semmelweis University, 1089 Budapest, Hungary; 3 Institute of Biology, ELTE Eötvös Loránd University, 1117 Budapest, Hungary; zsichla.levente@ttk.elte.hu; 4 National Laboratory for Health Security, ELTE Eötvös Loránd University, 1117 Budapest, Hungary

**Keywords:** Hantavirus, Dobrava-Belgrade virus, Puumala virus, RT-PCR, molecular diagnostics, phylogenetic analysis

## Abstract

Hantaviruses are globally distributed, rodent-borne zoonotic pathogens. In Hungary, Dobrava-Belgrade virus (DOBV) and Puumala virus (PUUV) are circulating, causing hemorrhagic fever with renal syndrome and nephropathia epidemica, respectively. Due to the short viremic period, hantaviruses are primarily diagnosed by serological methods. Detection of viral nucleic acid by real-time or nested reverse transcription polymerase chain reaction (RT-PCR) is limited to samples collected in the early phase of disease. Between 2018 and 2025, 51 laboratory-confirmed hantavirus infections were identified in Hungary; 30 cases were assigned to DOBV, 20 to PUUV, and one remained undetermined. Most patients were male (82%), suggesting increased exposure-related risk. Viral RNA was detected in 21 cases, mainly from serum and whole blood samples, and sporadically from urine. In three DOBV cases, viral RNA was detectable exclusively in whole blood but not in paired serum samples. Phylogenetic analysis included four PUUV and six DOBV partial S segment sequences showing high similarity to other human- and rodent-derived samples from the region. Hantavirus infections remain infrequently diagnosed in Hungary. Our findings suggest that serum and whole blood may be useful specimen types for molecular detection, whereas urine had limited diagnostic value in our dataset.

## 1. Introduction

Hantaviruses are enveloped, spherical-shaped, 80–120 nm diameter RNA viruses [1]. Their tri-segmented, negative-sense, single-stranded RNA genomes consist of a small (S), a medium-size (M), and a large (L) segment, which encode the viral nucleoprotein, envelope glycoprotein (Gn and Gc), and the RNA-dependent RNA polymerase, respectively [2]. Hantaviruses can infect endothelial, epithelial, and dendritic cells, as well as macrophages and lymphocytes, by binding to cell surface receptors via their viral glycoproteins [1].

According to the latest taxonomic classification, hantaviruses belong to the *Hantaviridae* family, order *Elliovirales*, and class *Bunyaviricetes*. The genus *Orthohantavirus* currently includes 38 species, including those that cause human diseases [3]. Based on geographic distribution, hantaviruses are divided into Old World and New World groups. Old World hantaviruses circulate in Eurasia and Africa and cause hemorrhagic fever with renal syndrome (HFRS) and the milder form of HFRS, nephropathia epidemica (NE). In contrast, New World hantaviruses are endemic in the Americas and cause hantavirus cardiopulmonary syndrome (HCPS) [4].

Rodents are the primary reservoirs, but hantaviruses have also been detected in lemmings, shrews, moles, and bats [4,5,6]. The virus persists asymptomatically in the hosts without causing disease, while infected rodents shed viruses in their excreta throughout their lives. Humans most commonly acquire infections through inhalation of aerosolized rodent excreta, but transmission can also occur via ingestion of contaminated food or rodent bites [5]. Human-to-human transmission has only been documented for the New World Andes virus (ANDV) [6].

In Europe, two Old World hantaviruses play a significant role in pathogenesis: Puumala virus (PUUV; *Orthohantavirus puumalaense*) and Dobrava-Belgrade virus (DOBV; *Orthohantavirus dobravaense*). The reservoir of the PUUV is the bank vole (*Myodes glareolus*), which is widespread in Europe. Most PUUV-associated diseases have been reported in Finland, Eastern Russia, and Sweden, but infections are also confirmed regularly in Germany, Belgium, France, Norway, Austria, and Hungary [7]. DOBV is endemic to Southeastern Europe, especially the Balkans, and carried by the yellow-necked wood mouse (*Apodemus flavicollis*) [7]. Additional genotypes of DOBV, including DOBV-Aa (Kurkino), Saaremaa virus (SAAV), and Sochi virus, are associated with *Apodemus agrarius* and *Apodemus ponticus* and exhibit regional distributions across Estonia, Germany, Slovakia, and Russia [8,9]. Subsequent serological and molecular analysis of hantavirus seroprevalence in humans and small mammals confirmed the circulation of at least two species, PUUV and DOBV, in Hungary [10]. Later, the presence of Tula virus and three DOBV genotypes was also reported in rodents [11,12,13].

This study aimed to summarize the epidemiological and laboratory findings of hantavirus infections in Hungary over the past eight years. Since there is limited data in the literature on the suitability of different sample types for hantavirus RNA detection, we also aimed to improve diagnostic practice by evaluating the feasibility of viral nucleic acid detection across various clinical specimens used in molecular diagnostics. As the National Reference Laboratory for Viral Zoonoses (National Center for Public Health and Pharmacy, Budapest, Hungary) is exclusively responsible for the laboratory diagnostics of human hantavirus infections in Hungary, this study provides a nationwide overview of the epidemiological and microbiological characteristics of human hantavirus infections.

## 2. Materials and Methods

### 2.1. Patients

Between 2018 and 2025, 1208 patients were tested for suspected hantavirus infections in the Hungarian National Reference Laboratory for Viral Zoonoses. Patients typically presented with non-specific symptoms (fever, headache, joint pain, gastrointestinal complaints) or with clinical signs of renal involvement, such as acute renal failure, nephritis, reduced kidney function, proteinuria, oliguria, or lumbar pain. A smaller number of requests were submitted for patients with pneumonia. Acute or recent hantavirus infection was confirmed in 51 patients with serological methods. Among the diagnosed cases, one was classified as an imported case (Romania), while the remaining cases had no documented travel history, suggesting local transmission within Hungary. All confirmed cases presented with either non-specific symptoms or renal manifestations; no cases associated with pneumonia were identified. According to the available information, more than half of the patients (29/51) were likely to have had rodent contact related to their occupation or living conditions. Reported professions included forestry and agricultural workers, hunters, carpenters, soldiers, police officers, gardeners, and a cheese factory worker.

### 2.2. Case Definition and Diagnostic Algorithm

The National Reference Laboratory for Viral Zoonoses applies a case definition for acute or recently acquired hantavirus infection based on the CDC case definition, with slightly more stringent criteria [14]. While the CDC case definition permits confirmation based on the detection of hantavirus-specific IgM antibodies or a rise in hantavirus-specific IgG titers alone, the case definition used in our laboratory requires at least one of the following:

- Detection of high titers of hantavirus-specific IgG antibodies in serum in the presence of IgM antibodies, or
- Seroconversion or at least a four-fold increase in the hantavirus-specific antibody titers in paired serum samples, or
- Detection of hantavirus-specific ribonucleic acid in clinical specimens.

During hantavirus diagnostics in immunocompetent patients, a stepwise diagnostic algorithm was applied. Serology was used as the primary screening method. Samples reactive for IgG and/or IgM antibodies by indirect immunofluorescence assay (IIFA) were further analyzed using an IgM immunoblot assay for confirmation and hantavirus typing. Hantavirus typing was performed primarily by immunoblot. IIFA results were used as supportive evidence by comparing virus-specific IgG antibody titers; a hantavirus species was assigned as the probable causative agent when the corresponding IgG titer was at least fourfold higher than those against other hantavirus species. Seroreactive samples were subsequently tested in parallel using PUUV- and DOBV-specific real-time RT-PCR (reverse transcription polymerase chain reaction) and nested RT-PCR assays. Nested PCR amplicons were subjected to Sanger sequencing for sequence confirmation and molecular characterization.

### 2.3. Serological Investigation

A virus-specific immune response was detected in human serum samples using in-house and commercially available indirect immunofluorescent assays (IIFAs) and immunoblot assay according to the manufacturer’s instructions. In-house IIFA was carried out as described previously in the literature [15], with minor modifications. In our laboratory, in-house PUUV- and DOBV-specific IIFAs were used. Since 2023, the commercially available Hantavirus Mosaic 1 IIFT test (Euroimmun Medizinische Labordiagnostika, Lübeck, Germany) has been used for routine diagnostics. For typing hantaviruses and to confirm acute infections, the Euroline Anti-Hanta Profile 1 IgM immunoblot assay (Euroimmun Medizinische Labordiagnostika, Lübeck, Germany) was performed. To avoid a false interpretation, in the case of early sampling, a second serum sample was collected from the same patient, on average 10–14 days later, compared to the first sampling.

### 2.4. Molecular Investigation

To detect hantavirus RNA, 44 serum, 17 EDTA-treated whole blood, and 18 urine samples from serologically confirmed patients were used, in a total of *n* = 44 patients. In the case of 7 confirmed patients, PCR testing was not performed because the samples were insufficient (3 patients) or were collected at a later phase of the disease (more than 10 days passed between symptom onset and sampling; 4 patients). Samples were stored at −80 °C until molecular testing. Total nucleic acid was extracted from 140 µL of the specimens using the QIAmp Viral RNA Mini Kit (QIAGEN, Hilden, Germany) according to the manufacturer’s instructions. Five µL of extracted RNA was reverse transcribed in a 20.5 µL final reaction volume. The reverse transcription reaction mixture consisted of the following reagents: 3 µL of Ultra Pure DNase/RNase-free distilled water (Invitrogen^TM^, Thermo Fisher Scientific, Waltham, MA, USA), 2 µL of 10× MuLV Reverse Transcriptase Buffer (New England BioLabs, Ipswich, MA, USA), 4 µL of 25 mM MgCl_2_ (Thermo Fisher Scientific, Waltham, MA, USA), 4 µL of dNTP mix (10 mM each) (Applied Biosystem, Thermo Fisher Scientific, Waltham, MA, USA), 1 µL of 50 µM random hexamer primers (Invitrogen^TM^, Thermo Fisher Scientific, Waltham, MA, USA), 0.5 µL of 200 U/µL MuLV Reverse Transcriptase (New England BioLabs, Ipswich, MA, USA) and 1 µL of 20 U/µL RNase Inhibitor (Applied Biosystem, Thermo Fisher Scientific, Waltham, MA, USA). The reverse transcription reaction was incubated at 25 °C for 5 min and 42 °C for 30 min, and enzyme inactivation was carried out at 99 °C for 5 min, followed by rapid cooling to 4 °C.

RT-PCR products were amplified using real-time and nested PCR protocols with the primer sets shown in Table 1. A TaqMan assay was performed using primer sets described earlier [16,17], with minor modifications specific to the S segment region of PUUV and DOBV hantaviruses (Puumala virus: primer pair PUF1, PUR1, and probe CRRSAp; Dobrava-Belgrade virus: primer pair DOBFP, DOBRP, and probe DOBP). The reaction was performed in a Light Cycler 2.0 instrument (Roche Life Science, Basel, Switzerland). The total reaction volume of 20 µL contained 10 µL of template cDNA, 3.5 µL of Ultra Pure DNase/RNase-free distilled water (Roche Life Science, Basel, Switzerland), 4 µL of ready-to-use hot start reaction mixture (LightCycler^®^TaqMan^®^Master, Roche Life Science, Basel, Switzerland), 1.25 pmol of each primer, and 0.25 pmol of the probe.

From 2024, the commercially available RealStar^®^ Hantavirus-HFRS RT-PCR kit 1.0 (Altona Diagnostics GmbH, Hamburg, Germany) was also performed to confirm the presence and typing of hantavirus RNA.

Viral RNA was also amplified using a *Hantavirus* genus-specific nested RT-PCR protocol with primer sets specific for the 397-nt region in the S segment, targeting the nucleocapsid gene. Degenerated primers published previously by Scharninghausen et al. were used for PCR reaction (outer primers: M-4 and M-9; internal primers: M-6 and M-8) [18]. The amplification reaction mixtures of both the first- and second-round PCR assays consisted of 5 μL of template DNA, 5.5 μL of Ultra Pure DNase/RNase-free distilled water (InvitrogenTM, Thermo Fisher Scientific, Waltham, MA, USA), 12.5 μL of ready-to-use 2× My Taq Red Mix (Bioline, Meridian Bioscience, Cincinnati, OH, USA), and 2.0 pmol of each primer. The nested PCR amplification profile was as follows. First round: initial denaturation was carried out at 94 °C for 2 min, followed by 5 cycles of 94 °C for 45 s, 45 °C for 30 s, 72 °C for 60 s, 30 cycles of 94 °C for 45 s, 50 °C for 30 s, 72 °C for 60 s, and the final extension step was carried out at 72 °C for 7 min; second round: initial denaturation was carried out at 94 °C for 2 min, followed by 5 cycles of 94 °C for 60 s, 62 °C for 60 s, 72 °C for 60 s, 5 cycles of 94 °C for 60 s, 58 °C for 60 s, 72 °C for 60 s, 25 cycles of 94 °C for 60 s, 58 °C for 60 s, and 72 °C for 60 s, and the final extension step was carried out at 72 °C for 10 min. PCR products were visualized in a 2% Tris-borate-EDTA agarose gel stained with ECO Safe (Pacific Image Electronics, Torrance, CA, USA).

Regarding the PCR quality control, besides a positive-template control, two negative controls are also included in each run: a nucleic acid extraction negative control and a no-template negative control for monitoring reagent contamination. In addition, the RealStar^®^ Hantavirus-HFRS RT-PCR kit 1.0 includes an internal control, which allows the detection of potential PCR inhibition.

To perform Sanger sequencing, nested PCR amplicons were purified using Viogene’s Advanced^TM^ PCR Clean Up System (Viogen Biotek Corporation, New Taipei City, China) following the manufacturer’s instructions. Direct sequencing of the amplicons was performed on a 3500 Genetic Analyzer (Applied Biosystems, Thermo Fisher Scientific, Waltham, MA, USA) using the BigDye^®^ terminator V3.1 cycle sequencing kit (Applied Biosystems, Thermo Fisher Scientific, Waltham, MA, USA) according to the manufacturer’s recommendations. Bidirectional sequencing of the DNA region of interest was performed using M-6 and M-8 primers. Nucleotide sequences were identified using the Basic Local Alignment Search Tool (BLAST, http://blast.ncbi.nlm.nih.gov/Blast.cgi; accessed on 15 December 2025). A phylogenetic maximum likelihood tree was created by MEGA 11 (Molecular Evolutionary Genetic Analysis) software (version 11) using ClustalW alignments of a 345-nucleotide-long partial sequence of the S segment nucleocapsid gene of hantaviruses. Bootstrap analysis of 1000 replicates was carried out to estimate the tree topology’s reliability. The evolutionary distance was calculated using the General Time Reversible model. The phylogenetic tree was rooted using the midpoint rooting method.

## 3. Results

### 3.1. Serological and Epidemiological Results

**Figure 1:**
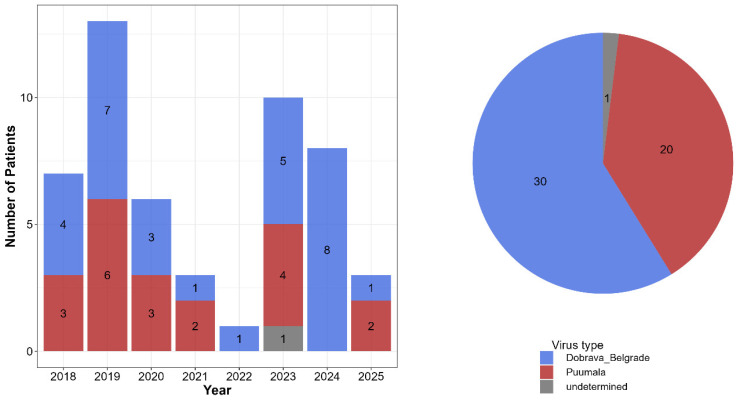
Annual number of laboratory-confirmed hantavirus infections and the proportional distribution of hantavirus species causing disease in Hungary between 2018 and 2025.

Between 2018 and 2025, 51 cases of hantavirus infection were diagnosed in Hungary by serological methods. All positive cases included in this study were confirmed by the high titers of hantavirus-specific IgG antibodies in the patients’ serum and detection of hantavirus-specific IgM antibodies by the immunoblot method. Figure 1 shows the annual number of laboratory-confirmed cases and the proportions of the causative hantavirus species. Dobrava-Belgrade virus (DOBV) accounted for 58.8% of cases (*n* = 30), while Puumala virus (PUUV) was responsible for 39.2% (*n* = 20). In one case, IgG titers were comparable for all tested hantavirus species, and no differences in band intensity were observed in the IgM immunoblots. PCR testing was negative; therefore, the infecting hantavirus species could not be identified in that case. Both PUUV and DOBV infections were detected in most years, except in 2022 and 2024, when only DOBV infections were confirmed (1 and 8 cases, respectively).

**Figure 2:**
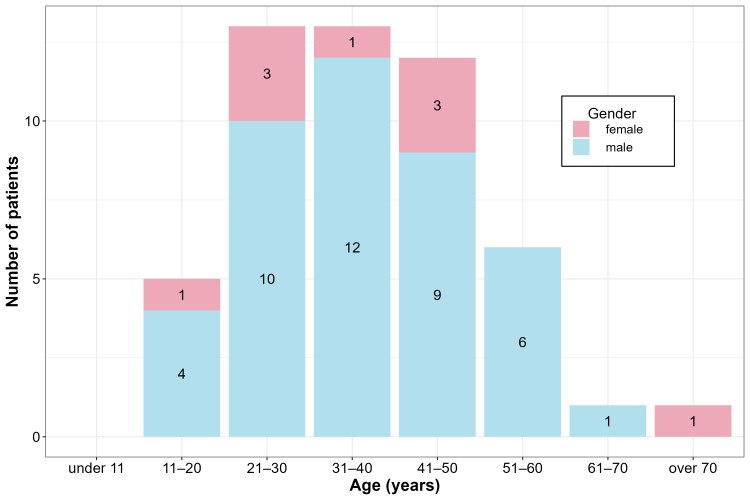
Age and gender distribution of patients diagnosed with hantavirus infection in Hungary between 2018 and 2025.

**Figure 3:**
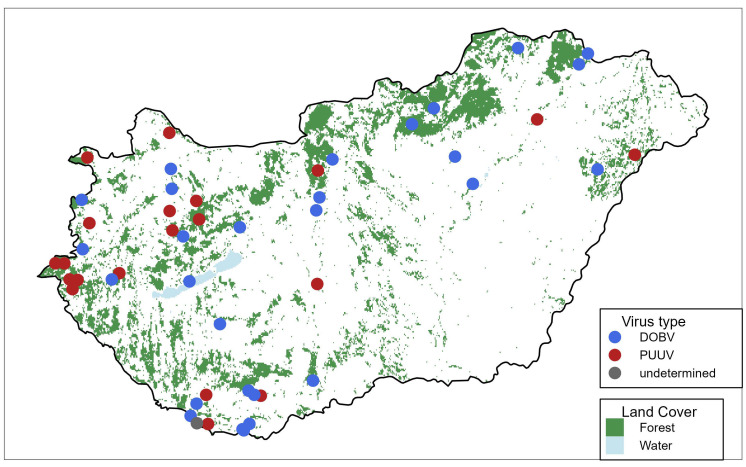
Geographical distribution of confirmed hantavirus cases in Hungary between 2018 and 2025. Blue symbols indicate Puumala virus (PUUV) infections, red symbols indicate Dobrava-Belgrade virus (DOBV) infections, and the gray symbol represents an undetermined case.

Among the patients examined in this study, hantavirus infection occurred mainly in males (82.3%, *n* = 42), with females representing 17.7% (*n* = 9). The average age of the infected patients was 37.6 years (range: 11–76) (Figure 2). An analysis of the spatial distribution of hantavirus infections by patients’ place of residence showed a pronounced predominance in rural and forested regions (including the Mecsek, Alpokalja, Transdanubian Mountains, and North-Hungarian Mountains). In contrast, only seven out of the 51 confirmed cases originated from major urban centers (Figure 3).

Regarding symptoms, the two leading symptoms in both Puumala and Dobrava infections were acute renal failure and fever. The clinical and laboratory characteristics of infected patients are listed in Table 2. Most of the symptoms listed occur with approximately the same frequency in individuals infected with PUUV and DOBV; however, certain symptoms, such as proteinuria, are more commonly observed in DOBV infections, whereas nephritis occurs at a higher rate in PUUV-infected patients.

**Table 2:** Clinical symptoms and laboratory parameters of patients with confirmed hantavirus infection (*n* = 51) in Hungary (2018–2025) based on the data provided on the examination request forms.

Clinical Symptom/Laboratory Parameter	Number of Cases	PUUV Infection *n* = 20 No. Patients (%)	DOBV Infection *n* = 30 No. Patients (%)
acute kidney failure/ decreased kidney function	36 *	13 (65%)	22 (73.3%)
fever	35	14 (70%)	21 (70%)
thrombocytopenia	11	4 (22.2%)	7 (24.1%)
low back pain	11	3 (16.6%)	8 (27.6%)
liver failure/ liver function abnormalities	9	4 (22.2%)	5 (17.2%)
diarrhea	9	2 (11.1%)	7 (24.1%)
nausea, vomiting	7	3 (16.6%)	4 (13.8%)
headache	7	3 (16.6%)	4 (13.8%)
proteinuria	7	1 (5.5%)	6 (20.7%)
nephritis	6	4 (22.2%)	2 (6.9%)
muscle-joint pain	5	1 (5.5%)	4 (13.8%)
oliguria	4	2 (11.1%)	2 (6.9%)
abdominal pain	3	2 (11.1%)	1 (3.4%)
hematuria	2	1 (5.5%)	1 (3.4%)
elevated CRP	2	0	2 (6.9%)
myopia	1	1 (5.5%)	0
ascites	1	1 (5.5%)	0
rash	1	1 (5.5%)	0
dizziness	1	1 (5.5%)	0

*: In the case of an undetermined hantavirus infection, the symptom was also acute kidney failure.

### 3.2. Molecular Findings and Phylogenetic Analysis

A total of 51 cases were confirmed by serological assays, of which 28 samples derived from 21 patients tested positive by PCR. Hantavirus RNA was detected in 18/44 serum samples, 7/17 EDTA-treated whole blood samples, and 3/18 urine samples. PUUV RNA was detected in 13 patients (14 samples: 13 serum and 1 whole blood), while DOBV RNA was detected in 8 patients (14 samples: 5 serum, 6 whole blood, and 3 urine). In three DOBV cases, viral RNA was detected exclusively in EDTA-treated whole blood but not in the corresponding serum. Urine samples tested positive only for DOBV infections and were detectable only by qPCR, with high Ct values (Ct > 37). Moreover, urine samples yielded positive PCR results only when concomitant serum or whole blood specimens from the same patient were also positive. Detailed results of viral RNA positivity distribution in different sample types and PCR methods are summarized in Table 3.

**Figure 4:**
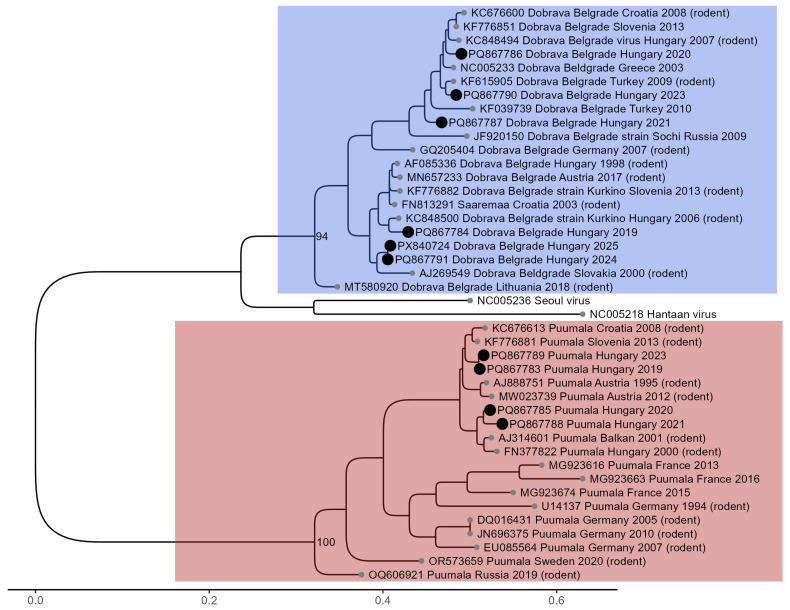
Maximum-likelihood phylogenetic tree of hantavirus sequences based on a 345-nucleotide fragment of the S segment nucleocapsid gene. Hungarian sequences obtained from serum samples of four patients infected with Puumala virus (GenBank accession numbers: PQ867783, PQ867785, PQ867788, and PQ867789), as well as from one serum sample (GenBank accession number: PQ867787) and five EDTA-treated whole blood samples (GenBank accession numbers: PQ867784, PQ867786, PQ867790, PQ867791 and PX840724) of six patients infected with Dobrava-Belgrade virus, are indicated by black circles. The blue rectangle represents DOBV sequences, the red rectangle represents PUUV sequences. The numbered line at the bottom indicates evolutionary distance. Hantavirus sequences are labeled with the GenBank accession number, virus type, country of origin, and year of isolation or submission (if available). Sequences derived from rodent hosts are marked in brackets.

Thirteen out of twenty-one samples obtained from thirteen patients were available for subsequent analysis by Sanger sequencing. Three PUUV-positive serum samples yielded only shorter partial nucleocapsid gene sequences; these were excluded from phylogenetic analysis but are provided in the Supplementary Materials. Phylogenetic analysis was performed on PUUV sequences obtained from four serum samples and on DOBV sequences from one serum sample and five whole-blood samples (Figure 4). All sequences generated in this study are available under GenBank accession numbers PQ867783-91 and PX840724. The obtained PUUV sequences exhibited 92–96% sequence identity with hantavirus strains from Austria, Slovenia, and Croatia (GenBank accession numbers: MW023739, AJ888751, KF776881, KC676600). Our DOBV sequences fall into two separate branches of the phylogenetic tree. Three Hungarian DOBV sequences (PQ867786-87, PQ867790) clustered with Turkish, Greek, Croatian, and Slovenian strains (GenBank accession numbers: KF039739, KC848500, NC005233, KC676600, KF776851). Another three DOBV sequences (PQ867784, PQ86791, and PX840724) clustered with strains reported from Slovakia, Croatia, Slovenia, and Austria (GenBank accession numbers: AJ269549, FN813291, KF776882, MN657233). Previously reported Hungarian sequences obtained from rodents showed a high degree of nucleotide similarity with those derived from human cases (GenBank accession numbers: FN377822, KC848494, KC848500). The PUUV sequences generated in this study shared 94–96% pairwise nucleotide identity with FN377822. In the case of DOBV, the sequences PQ867786 and KC848494 were 98% identical, whereas PQ867784 and KC848500 showed 97% identity.

## 4. Discussion

In this study, we provide an updated overview of hantavirus infections in Hungary between 2018 and 2025, including epidemiological, clinical, and molecular data. Hantavirus infections in Hungary were predominantly caused by DOBV, with PUUV also circulating during most years, and mainly affected middle-aged males from rural and forested regions. Clinical manifestations were broadly comparable between PUUV and DOBV infections, with renal failure and fever as the most common symptoms. Molecular and phylogenetic analyses revealed sample-type-dependent RNA detectability and demonstrated that Hungarian strains cluster with Central and Southeastern European hantavirus lineages.

**Figure 5:**
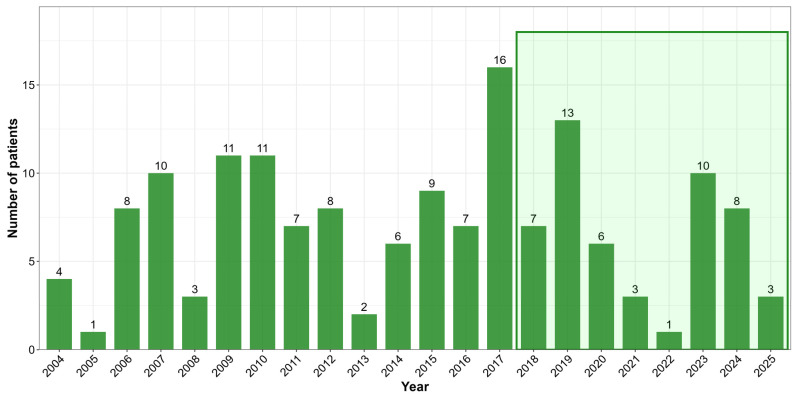
The annual number of laboratory-diagnosed hantavirus cases in Hungary, between 2004 and 2025 (based on the data of the National Center for Public Health and Pharmacy, Budapest, Hungary). The framed part represents the data of the period discussed in this study.

Hantavirus infection has been a notifiable disease in Hungary for several decades. Long-term surveillance data indicate sustained circulation of hantaviruses in both human and rodent populations [10,11,19]. Although hantavirus-associated renal syndrome was already observed among soldiers in the early 1950s, laboratory-confirmed HFRS cases were first reported in 1971 by Trencsényi et al. [20]. Routine serological diagnostics for hantavirus infections were introduced in the early 1980s. Previous seroepidemiological investigations among Hungarian forestry workers demonstrated a seroprevalence of 4.6%, indicating occupational exposure risk [21]. Serological surveys in small mammals further confirmed the endemic circulation of hantaviruses, supporting the zoonotic nature of human infections, and estimated a hantavirus antibody prevalence of 7.25% [10,12]. Temporal trends in hantavirus case numbers observed between 2004 and 2017 were broadly consistent with those identified in the current study period (Figure 5; based on the data of the National Center for Public Health and Pharmacy, Budapest, Hungary). The present findings reinforce earlier observations that hantavirus infections in Hungary show a strong spatial association with forested and mountainous regions, which are considered high-risk environments for the virus transmission. In addition, the disproportionate involvement of younger adults observed in our study is likely reflected in increased occupational and recreational exposure.

At the European level, a recent systematic review and meta-analysis of 33 studies estimated the overall hantavirus seroprevalence in Europe at 2.98% [22]. Surveillance data from 28 European countries, published by the European Centre for Disease Prevention and Control (ECDC), indicate that Finland and Germany account for the majority (60.5–85%) of reported cases, predominantly caused by the PUUV [23,24]. The male predominance and higher incidence among individuals older than 25 years reported across Europe are consistent with our observations, suggesting shared exposure-related risk factors [23,24]. Comparison of reported hantavirus infections in neighboring countries reveals that annual case numbers in the Czech Republic, Bulgaria, and Romania are comparable to those in Hungary. In contrast, Croatia, Austria, Slovenia, and Slovakia consistently report higher annual incidence [23,24].

In Central Europe and the Balkans, PUUV and DOBV are the predominant causative agents of HFRS. Bosnia and Herzegovina is a highly endemic area with circulating DOBV, PUUV, and Seoul virus (SEOV), where 26.1% and 49.6% of infections are attributed to DOBV and PUUV, respectively [25]. In the Czech Republic, the human seroprevalence of hantaviruses ranges from 1.0% to 1.4%, with both DOBV and PUUV circulating [26]. PUUV predominates in Slovenia, Croatia, and Austria [27,28,29,30], whereas in Bulgaria, 69.6% of HFRS cases were attributed to DOBV and 30.4% to PUUV [31]. In Serbia, DOBV has been identified as the primary causative agent of HFRS [32] while in Romania, only DOBV infections have been reported [27]. The results of the present study align well with published data from Central European countries, further supporting the epidemiological relevance of PUUV and DOBV in Hungary. However, serology-based hantavirus typing should be interpreted with caution, as cross-reactivity between hantavirus species may affect virus assignment, particularly in cases without molecular confirmation. In our study, such cross-reactivity was observed with both IIFA and immunoblot, although the immunoblot assay appeared to be more specific than IIFA. Immunoblot has been reported to allow reliable identification of the infecting hantavirus, even though serology-based typing may still be influenced by cross-reactivity in some cases [33]. In our study, no discrepancies were observed between serological and molecular typing in PCR-positive cases.

Data on the suitability of different clinical specimen types for the molecular detection and characterization of DOBV and PUUV are limited. Most studies have relied on serum samples for hantavirus RNA detection [32,34], whereas whole blood and urine have been used only sporadically, with their diagnostic utility remaining uncertain [26,31]. A study conducted in France evaluated the presence of PUUV RNA in plasma and urine samples, concluding that urine is not an appropriate specimen for molecular diagnostics [35], a result consistent with our findings. Although the relatively small number of cases precludes firm conclusions, our observations raise the possibility that whole blood may be a more suitable sample type than serum for DOBV RNA detection. To our knowledge, the potential suitability of whole blood over serum for DOBV RNA detection has not been previously described. Therefore, this assumption requires further studies conducted on a larger sample size.

In addition to sample type, methodological differences between the applied molecular assays may also have influenced RNA detectability. The lower diagnostic sensitivity of the nested PCR assay observed in our study may be related not only to primer specificity but also to differences in amplicon length between the two molecular methods. Compared with the real-time PCR assay, the nested PCR targets a substantially longer genomic region, which may reduce detectability in samples with partially degraded or fragmented nucleic acid. Under such conditions, shorter targets are more likely to remain detectable than longer ones.

## 5. Conclusions

In summary, our data support the continued circulation of PUUV and DOBV in Hungary, consistent with regional epidemiological patterns in Central Europe. Our results suggest that specimen type may influence the performance of molecular hantavirus diagnostics, with EDTA-anticoagulated whole blood potentially offering advantages for DOBV RNA detection, whereas urine samples appeared to have limited diagnostic utility in our dataset. Larger, systematically designed studies are needed to confirm these findings and to inform the harmonization of molecular diagnostic and sampling strategies in endemic settings.

## 6. Limitations

This study has some limitations, which may have influenced the interpretation of the results. These are partly related to the nature of a diagnostic laboratory setting, where conclusions must be drawn from the available information and the patient samples accessible for analysis. The following limitations of this study should be acknowledged: small sample size, availability of the different sample types from the same patient, the inability to systematically assess the exact time since symptom onset, and the restricted clinical/anamnestic information available from test request forms. As a reference laboratory, we do not have direct contact with patients or clinicians, and therefore, our analysis was limited to the data provided with the submitted samples. In addition, the observed male predominance reflects only the confirmed cases included in this study and should not be interpreted as a population-level estimate.

## Figures and Tables

**Table 1:** Oligonucleotide primers and TaqMan probes used in this study.

Primers Designation	Sequences 5′–3′
S segment real-time RT-PCR *Puumala virus*	
PUF1	GAAAA**R**AACTGG**A**ATGAGTGACTTG *
PUR1	TT**R**AG**T**TTTTGTCTGGCAACA *
CRRSAp	/HEX/ATAACCCGCCATGAACAACAGCTTG/BHQ2/
*Dobrava-Belgrade virus*	
DOBFP	TGGCTTGACCT**Y**CC**R**TG *
DOBRP	CAAG**Y**GCTCCTTGTCTTTG**R** *
DOBP	/FAM/ATCTCCAACGTCTTTGACCAAAGGCCC/TAMRA/
S segment nested RT-PCR	
M-4	ATGAARGCNGAWGARNTNACMCCNGG
M-9	TGRYCNAGYTGTATYCCCATWGATTG
M-6	AGYCCWGTNATGRGWGTRATTGG
M-8	GAKGCCATRATNGTRTTYCKCATRTCCTG

* The bases marked in bold represent the modification compared to the original sequences.

**Table 3:** Summary of hantavirus RNS detection results in different sample types using real-time RT-PCR and nested RT-PCR methods (2018–2025).

Specimen Type	Number of Tested Samples	Number of PCR-Positive Samples (Any Method) *	Number of Real-Time RT-PCR-Positive Samples ^#^	Number of Nested RT-PCR-Positive Samples	Number of Samples Positive by Both Real-Time RT-PCR and Nested RT-PCR
Serum	44	18	16	11	9
EDTA-treated whole blood	17	7	5	6	4
Urine	18	3	3	0	0
**Total**	**79**	**28**	**24**	**17**	**13**
					
*Puumala* *virus*					
Serum	17	13	12	7	6
EDTA-treated whole blood	5	1	1	0	0
Urine	7	0	0	0	0
**Total**	**29**	**14**	**13**	**7**	**6**
					
*Dobrava-Belgrade virus*					
Serum	27	5	4	4	3
EDTA-treated whole blood	12	6	4	6	4
Urine	11	3	3	0	0
**Total**	**50**	**14**	**11**	**10**	**7**

* PCR-positive samples indicate positivity by at least one PCR method described in the Materials and Methods section. # Real-time PCR results with Ct values of up to 40 are considered positive, whereas samples with Ct values above 40 are re-analyzed and interpreted as positive only if confirmation is obtained by nested PCR. Gray shading indicates the results for all samples analyzed. EDTA: ethylenediaminetetraacetic acid; RT-PCR: reverse transcription polymerase chain reaction.

## Data Availability

Data are contained within the article or Supplementary Materials.

## Supplementary Materials

The following supporting information can be downloaded at: https://www.mdpi.com/article/10.3390/v18030366/s1, Supplementary Materials: Detailed results of partial sequences of Puumala virus.

## Abbreviations

The following abbreviations are used in this manuscript:

ANDV	Andes virus
DOBV	Dobrava-Belgrade virus
EDTA	Ethylenediaminetetraacetic acid
HCPS	Hantavirus cardiopulmonary syndrome
HFRS	Hemorrhagic fever with renal syndrome
IIFA	Indirect immunofluorescence assay
NE	Nephropathia epidemica
PUUV	Puumala virus
RNA	Ribonucleic Acid
RT-PCR	Reverse Transcription Polymerase Chain Reaction
SAAV	Saaremaa virus
SEOV	Seoul virus
