# Supplementary material for: Human Hantavirus Infections in Hungary (2018–2025): Epidemiology, Molecular Detection Across Clinical Sample Types, and Phylogenetic Analysis
Source: Viruses. 2026 Mar 16;18(3):366. doi: 10.3390/v18030366 (PMC13030873; doi:10.3390/v18030366)
Supplement: Supplementary file 1 [file viruses-18-00366-s001.zip › viruses-4188998-supplementary.pdf]

>6221_2018_PUUV_serum_316bp
TCTTTTTTTGTAAAGGATTGGTCAGAGAGGGTAAGAGATTTTATGGAGAAAGAATGCCCTTTTATAAAACCAG
AGATTAAACCAGGGACACCCCCACAGGAGGCTGAGTTCTTAAAAAGAAATAAAGTGTACTTCATGCAGCGTCA
AGATGTCCTAGATAAAAATCATGTGGCAGACATTGATAAGTTAATTGATTATGCAGCCTCAGGGGACCCTACTT
CACCTGACAACATAGAATCCCCTAATGCACCATGGGTATTTGCATGTGCTCCAGATCGGTGCCCACCGACATGT
ATCTTATGTGCAGGGATGGCGA

>3679_2019_PUUV_serum_317bp
TTCTCTTTCTTTGTAAAGGATTGGTCAGAAAGGGTAAGAGAATTTATGGAGAAAGAATGCCCCTTTATAAAACC
AGAGATTAAGCCAGGGACACCCCCACAGGAGGCAGAGTTCTTAAAAAGAAATAAAGTGTACTTCATGCAGCG
TCAAGATGTGCTAGATAAAAATCATGTGGCAGATATTGATAAGTTGATTGATTATGCAGCCTCAGGGGACCCA
ACATCACCTGACAACATAGAATCCCCTAATGCACCATGGGTATTCGCATGTGCTCCAGATAGGTGTCCACCAAC
ATGCATCTATGTTGCCAGGGATGG

>3976_2019_PUUV_serum_318bp
TTCTCTTTTTTTGTAAAAGATTGGTCAGAGAGGGTAAGAGAATTTATGGAGAAAGAATGCCCTTTTATAAAACC
AGAGATCAAACCAGGGACACCCCCGCAGGAGGCTGAGTTCTTAAAAAGAAATAAAGTGTACTTCATGCAGCG
TCAAGATGTGCTAGATAAAAATCATGTGGCAGATATTGATAAGTTAATTGATTATGCAGCCTCAGGGGACCCA
ACATCACCTGACWACATAGAATCCCCTAATGCACCATGGGTATTCGCAGGTGCCCCAGATAGGTGTCCMCCAA
CATGCATSTATGTTGCARGGGATGGT
